# Hydrodistillation and Microwave Extraction of Volatile Compounds: Comparing Data for Twenty-One *Veronica* Species from Different Habitats

**DOI:** 10.3390/plants11070902

**Published:** 2022-03-28

**Authors:** Valerija Dunkić, Marija Nazlić, Mirko Ruščić, Elma Vuko, Karla Akrap, Snježana Topić, Milenko Milović, Nenad Vuletić, Jasna Puizina, Renata Jurišić Grubešić, Siniša Srečec, Dario Kremer

**Affiliations:** 1 Faculty of Science, University of Split, Rudera Boškovića 33, 21000 Split, Croatia; dunkic@pmfst.hr (V.D.); mnazlic@pmfst.hr (M.N.); mrus@pmfst.hr (M.R.); elma@pmfst.hr (E.V.); kakrap@pmfst.hr (K.A.); topic@pmfst.hr (S.T.); nenov@pmfst.hr (N.V.); puizina@pmfst.hr (J.P.); 2 Antun Vrančić Grammar School, Put Gimnazije 64, 22000 Šibenik, Croatia; milenko.milovic@skole.hr; 3 Faculty of Medicine, University of Rijeka, Braće Branchetta 20, 51000 Rijeka, Croatia; renatajg@medri.uniri.hr; 4 Križevci College of Agriculture, Milislava Demerca 1, 48260 Križevci, Croatia; ssrecec@vguk.hr; 5 Faculty of Pharmacy and Biochemistry, University of Zagreb, A. Kovačića 1, 10000 Zagreb, Croatia

**Keywords:** *Veronica*, volatile compounds, microwave extraction, GC-MS, hexahydrofarnesyl acetone, hexadecanoic acid, phytol

## Abstract

Free volatile compounds were isolated from 21 Croatian *Veronica* species studied by hydrodistillation (HD) and microwave extraction (ME) and analyzed by gas chromatography coupled with mass spectrometry. Principal Component Analysis (PCA) distinguished some clusters based on the relative proportion of major compounds, such as hexadecanoic acid, hexahydrofarnesyl acetone, phytol, *E*-caryophyllene, and caryophyllene oxide, which were identified in all species studied by both isolation methods. In addition to these compounds, germacrene D, δ-selinene, and eicosane were also identified in five samples from dry habitats isolated using ME. *Allo*-aromadendrene and β-ionone are particularly abundant in five species from wet habitats isolated by both methods. The peculiarities of *Veronica* species from moderate habitats isolated with HD are benzene acetaldehyde, *n*-nonanal, and the identification of significant compounds from the hydrocarbon class, while the peculiarity of ME is (*E*)-β-damascenone. In this article, we present new results on the phytochemical characterization of *Veronica* species from different habitats. The biological potential of these compounds should be further investigated for a better understanding and utilization of the specialized plant metabolites.

## 1. Introduction

The genus *Veronica* L. from the family Plantaginaceae (formerly Scrophulariaceae) (order Lamiales) includes about 500 species that are distributed slightly more over the Northern Hemisphere [1]. A significant number of species (approximately 180) with the *Hebe* complex are spread throughout the Southern Hemisphere, i.e., New Zealand, Australia, and New Guinea [2]. Sixty-two *Veronica* species have been described for Europe [3] and thirty-seven for Croatia [4]. *Veronica* species are characterized by extreme variability in morphology, life forms, and habitats [2]. The ability to adapt to different living conditions has allowed these species to inhabit a variety of habitats, from aquatic and wetland habitats to rocky and dry habitats [2,5,6]. Most representatives of the genus *Veronica* grow in areas with a Mediterranean climate and from the sea level to high alpine regions [2]. *Veronica* species are herbs with a sometimes-woody stock and opposite low leaves. The floral leaves usually alternate. Solitary flowers develop in the leaf-axils or are arranged in axillary or terminal racemes. The calyx is divided into four or five, often unequal, segments. The corolla is rotated to the campanulate and different in color. There are two exerted stamens and one pistil in the flowers. The fruit is a capsule [3].

It is well known that plants are exposed to many environmental stresses, such as droughts, extreme temperatures, nutrient deficiencies, fires, flooding, salinity, excessive amounts of heavy metals, insect attacks, and various pathogenic microorganisms. These stresses affect their growth, development, and reproduction. As a response to environment stresses, weather conditions, and pathogen attacks, they produce specialized metabolites [7]. Tan and Nishida [8] found that phenylpropanoids, such as methyl eugenol and Z-methyl isoeugenol, occur in plants under the influence of pathogen attacks and ultraviolet radiation.

Phytochemical studies on species of the genus *Veronica* have mostly been focused on the content of glycosides, phenols, and flavonoids [9,10,11,12,13,14,15,16,17]. The free volatile compounds (VCs) of the *Veronica* species, on the other hand, have been much less studied [6,14,18,19,20,21]. One of the reasons for this is that research on VCs has mainly focused on plant families that are commonly known to be rich sources of these compounds, such as Lamiaceae, Geraniaceae, Asteraceae, Rutaceae, Lauraceae, and Myrtaceae.

The isolation of free VCs, which are important specialized metabolites of plants, can be done by classical and green extraction. Classical extraction techniques include steam distillation, hydro-diffusion, hydrodistillation, destructive distillation, and cold pressing. Green extraction techniques include turbo distillation, ultrasonic-assisted extraction, microwave-assisted extraction, and instant controlled pressure drop technology. Depending on the isolation technique, the composition of the essential oil extracted from the same plant material may vary. This is influenced by the duration of the distillation, the temperature, the pressure, and the quality of the plant material. Green extraction requires less time and less water than traditional extraction [22].

In this paper, we describe the phytochemical characterization of free VCs obtained by classical hydrodistillation (HD) and green microwave extraction (ME) from twenty-one *Veronica* species distributed in Croatia. The studied species of the genus *Veronica* were grouped by habitat so that the results could be easily summarized. The aim of this work was to obtain new data on the characterization of VCs as specialized metabolites. Qualitative and quantitative differences in the composition of these compounds can be identified using different isolation techniques, and the identification of these differences is very important for further biological research. For most of the studied *Veronica* species, data on the composition of volatile compounds are presented for the first time.

## 2. Results

### 2.1. Investigation of Veronica Species

Twenty-one species of the genus *Veronica* collected through field research were classified into three groups of habitats based on the general humidity of the habitat (dry, wet, and moderate). VCs isolated by classical HD and green ME were analyzed by gas chromatography (GC) coupled with mass spectrometry (MS) and the results are shown in Supplementary Tables S1–S6. In Tables S1–S6, the identified compounds are arranged according to the time of occurrence on the nonpolar capillary column (VF5-ms) and according to the eight corresponding classes (monoterpene hydrocarbons, oxygenated monoterpenes, sesquiterpene hydrocarbons, oxygenated sesquiterpenes, oxygenated diterpenes, phenolic compounds, hydrocarbons, and a common group of acids, alcohols, and esters).

### 2.2. Volatile Compounds of Veronica Species from Dry Habitats

**Figure 1:**
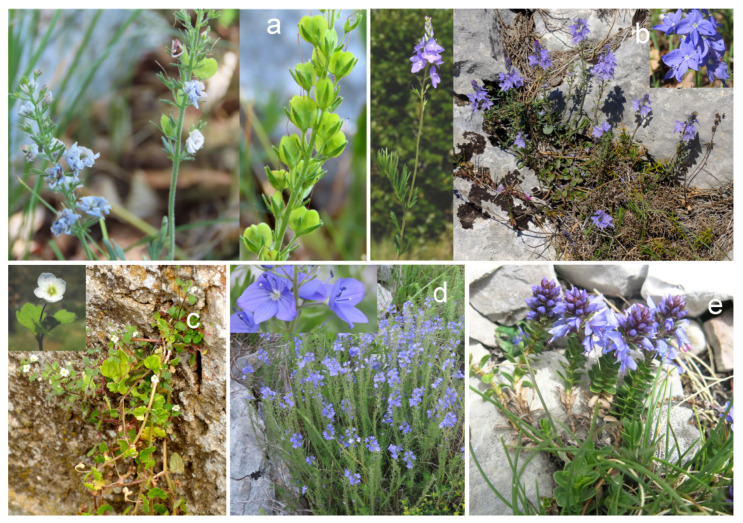
Photographs of *Veronica* taxa collected from dry habitats: *V. austriaca* ssp. *austriaca* (**a**), *V. austriaca* ssp. *jacquinii* (**b**), *V. cymbalaria* (**c**), *V. dalmatica* (**d**), and *V. saturejoides* ssp. *saturejoides* (**e**).

**Figure 2:**
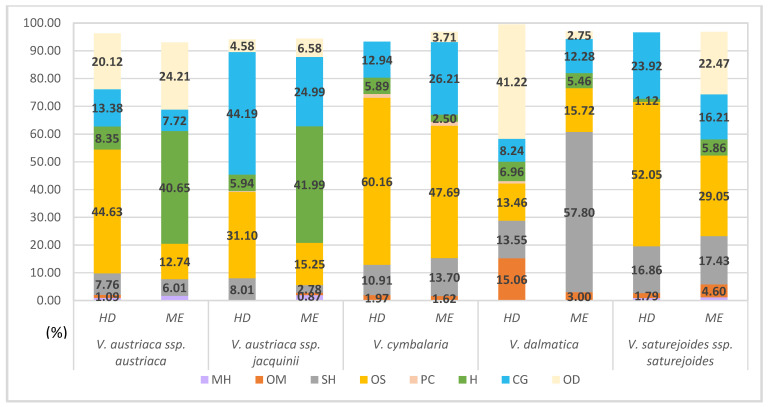
Relative content of volatile compounds in *Veronica* taxa collected from dry habitats. HD, hydrodistillation; ME, microwave extraction; MH, monoterpene hydrocarbons; OM, oxygenated monoterpenes; SH, sesquiterpene hydrocarbons; OS, oxygenated sesquiterpenes; PC, phenolic compounds; H, hydrocarbons; CG, common group of acids, alcohols, and esters; OD, oxygenated diterpenes.

The five *Veronica* species (*V. austriaca* L. ssp. *austriaca, V. austriaca* ssp. *jacquinii* (Baumg.) Eb. Fisch., *V. cymbalaria* Bodard, *V. dalmatica* Padilla-García, Rojas-Andrés, López-González et M. M. Mart. Ort., and *V. saturejoides* Vis. ssp. *saturejoides*) from dry habitats were studied (Figure 1). The term ‘dry habitat’ encompasses *Veronica* species that grow in open, rocky, sunny, or dry areas. The compound classes shown in Figure 2 for these studied species are listed according to Supplementary Tables S1 and S2, which compare the two isolation methods. The main classes with a percentage of identification greater than 50% are oxygenated sesquiterpenes with 60.16% in *V. cymbalaria* obtained by HD and sesquiterpene hydrocarbons with 57.8% in *V. dalmatica* obtained by ME (Figure 2).

The main constituents in the composition of VCs obtained by HD (Supplementary Table S1) are the following: The oxygenated sesquiterpene hexahydrofarnesyl acetone is the most abundant compound identified in *V. austriaca* ssp. *austriaca* (39.77%) and *V. cymbalaria* (36.33%). Hexadecanoic acid is the most abundant compound in *V. austriaca* ssp. *jacquinii* (32.17%). In the composition of the endemic species *V. dalmatica,* the oxygenated diterpene phytol is the most abundant with a percentage of identification of 41.22% (Figure 2), while in the composition of *V. saturejoides* ssp. *saturejoides* caryophyllene oxide is the most abundant (34.53%).

In the composition of VCs obtained via ME, phytol is the most abundant compound identified in *V. austriaca* ssp. *austriaca* (24.21%) and in *V. saturejoides* ssp. *saturejoides* (22.47%), while hexadecanoic acid is the most abundant compound identified in *V. austriaca* ssp. *jacquinii* (22.12%) (Figure 2; Supplementary Table S2). Caryophyllene oxide is the major compound in *V. cymbalaria* (32.72%), while *E*-caryophyllene is the most abundant compound in *V. dalmatica* (39.53%).

### 2.3. Volatile Compounds of Veronica Species from Wet Habitats

**Figure 3:**
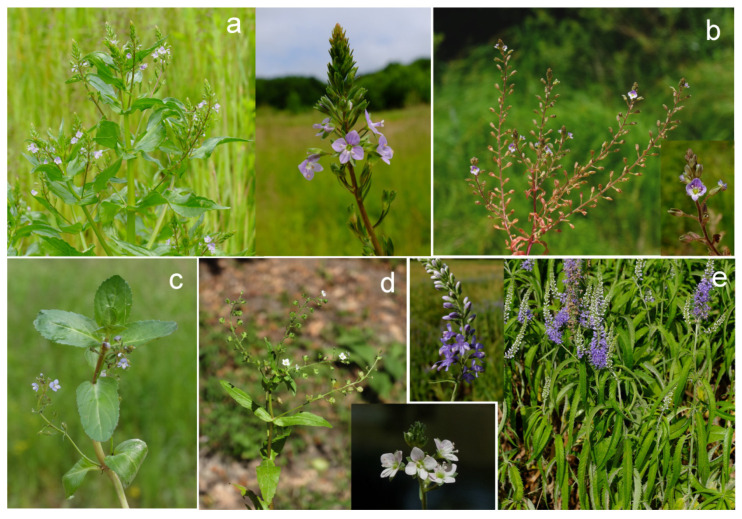
Photographs of *Veronica* taxa collected from wet habitats: *V*. *anagallis-aquatica* ssp. *anagallis*-*aquatica* (**a**), *V. anagalloides* (**b**), *V. beccabunga* (**c**), *V*. *catenata* (**d**), and *V. longifolia* (**e**).

**Figure 4:**
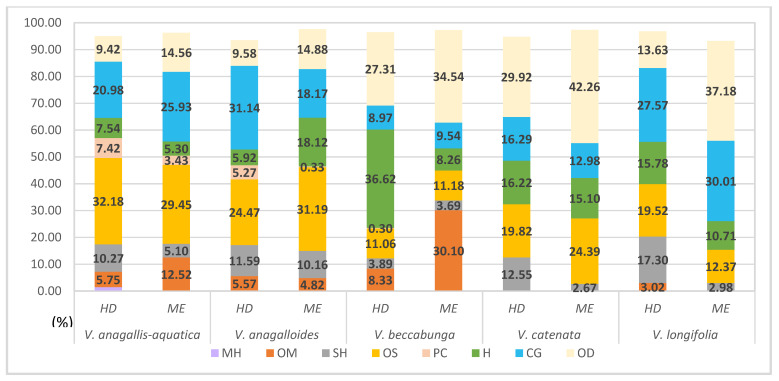
Relative content of volatile compounds in *Veronica* taxa collected from wet habitats. MH, monoterpene hydrocarbons; OM, oxygenated monoterpenes; SH, sesquiterpene hydrocarbons; OS, oxygenated sesquiterpenes; PC, phenolic compounds; H, hydrocarbons; CG, common group of acids, alcohols, and esters; OD, oxygenated diterpenes.

Five *Veronica* species (*V. anagallis-aquatica* L., *V. anagalloides* Guss., *V. beccabunga* L., *V. catenata* Pennell, and *V. longifolia* L.) were also collected from wet habitats (Figure 3). The term ‘wet habitat’ encompasses species that grow in lake or stream water. The compound classes for these studied species are shown in Figure 4, which compares the two isolation methods. Hexahydrofarnesyl acetone is the major compound in *V. anagallis-aqua*tica isolated by HD and ME (27.17% and 25.97%, respectively) (Supplementary Tables S3 and S4). It is also the predominant compound in *V. anagalloides* identified by both extraction methods (14.33% for HD and 19.12% for ME). In the same species, hexadecanoic acid was identified with a significant percentage (13.67%) by HD. The oxygenated diterpene phytol is the dominant compound in *V. beccabunga* (27.31% for HD and 34.54% for ME), *V. catenata* (29.92% for HD and 42.26% for ME), and *V. longifolia* (13.63% for HD and 37.18% for ME) isolated by both methods. *E*-caryophyllene, caryophyllene oxide, hexahydrofarnesyl acetone, phytol, hexadecanoic acid, and β-ionone were identified in all five *Veronica* species collected from wetland habitats. Monoterpene hydrocarbons were not isolated during green extraction in *Veronica* species from wet habitats (Supplementary Table S4).

### 2.4. Volatile Compounds of Veronica Species from Moderate Habitats

**Figure 5:**
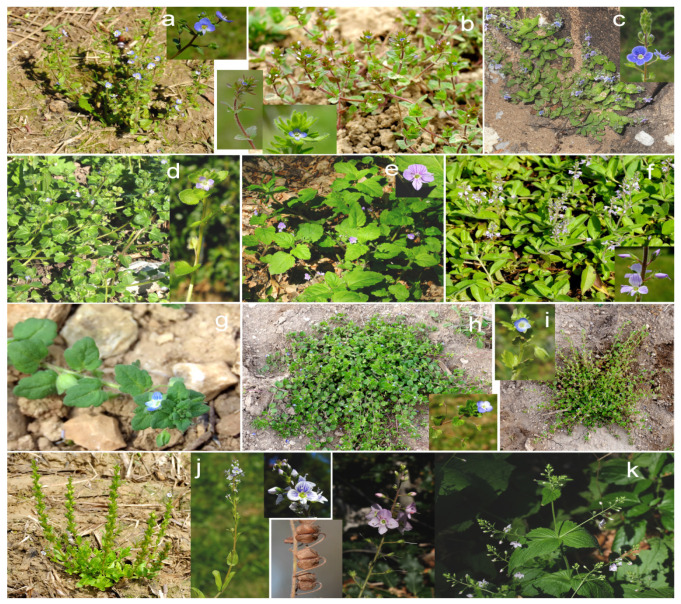
Photographs of Veronica species collected from moderate habitats: *V. acinifolia* (**a**), *V. arvensis* (**b**), *V. chamaedrys* (**c**), *V. hederifolia* (**d**), *V.* *montana* (**e**), *V. officinalis* (**f**), *V. opaca* (**g**), *V. persica* (**h**), *V. polita* (**i**), *V.* *serpyllifolia* (**j**), and *V.* *urticifolia* (**k**).

**Figure 6:**
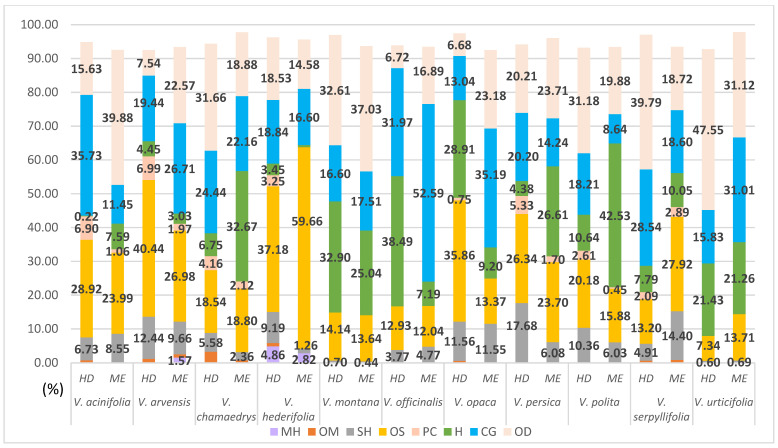
Relative content of volatile compounds in *Veronica* species collected from moderate habitats. MH, monoterpene hydrocarbons; OM, oxygenated monoterpenes; SH, sesquiterpene hydrocarbons; OS, oxygenated sesquiterpenes; PC, phenolic compounds; H, hydrocarbons; CG, common group of acids, alcohols, and esters; OD, oxygenated diterpenes.

Eleven species of the genus *Veronica* (*V. acinifolia* L., *V. arvensis* L., *V. chamaedrys* L., *V. hederifolia* L., *V. montana* L., *V. officinalis* L., *V. opaca* Fr., *V. persica* Poir., *V. polita* Fr., *V. serpyllifolia* L., and *V. urticifolia* Jacq.) were collected from moderate habitats (Figure 5). The term ‘moderate habitat’ encompasses Veronica species that grow in vineyards (*V. acinifolia*), orchards (*V. chamaedrys*), arable land (*V. arvensis*, *V. hederifolia*, *V. opaca*, *V. persica*, *V. polita*, and *V. serpyllifolia*), and mesophilic beech forests (*V. montana*, *V. officinalis*, and *V. urticifolia*). The isolation of the VCs was also carried out by both methods. The compound classes for the studied species are shown in Figure 6, which compares the two isolation methods. *E*-caryophyllene, caryophyllene oxide, hexahydrofarnesyl acetone, phytol, β-ionone, hexadecanoic acid, and docosane were identified in all eleven isolates obtained by HD (Supplementary Table S5). *V.* *acinifolia* is rich in β-ionone (17.01%), hexahydrofarnesyl acetone (15.37%), and caryophyllene oxide (7.71%). Hexahydrofarnesyl acetone (28.85%) is the most abundant compound in the composition of VCs in *V. hederifolia*. Additionally, the monoterpene hydrocarbon α-thujene (4.86%) was identified only in *V. hederifolia*. A peculiarity is the identification of γ-eudesmol in a high percentage (19.98%) only in *V. arvensis*. *V. chamaedrys*, *V. montana*, *V. persica*, *V. polita*, *V. serpyllifolia*, and *V. urticifolia* are rich in phytol (particularly *V. urticifolia* with an identification percentage of 47.55%). These species are also rich in heptacosane, as is *V. officinalis* with an identification percentage of 20.67%. Heptacosane, *E*-caryophyllene, and caryophyllene oxide were also the most abundant compounds in the composition of VCs obtained using HD from *V. opaca* with percentages greater than 11% (Supplementary Table S5).

The same major components identified in the isolates using HD are also predominant in the composition of the VCs isolated by ME in the 11 *Veronica* species collected from moderate habitats (Supplementary Table S6). The distinctive features are the high percentage of the hydrocarbon pentacosane (14.9%) identified in *V. montana* and the high percentage of heptacosane identified in *V. persica* and *V. polita* (14.28% and 15.13%, respectively). Another feature is that the isolate obtained by green extraction from *V. polita* is rich in octacosane (16.95%). The compound 3-hexen-*1-ol* was identified (22.04%) only in *V. officinalis* from isolates obtained by ME, and this component was not identified in other *Veronica* species studied either by classical or green extraction.

### 2.5. Principal Component Analysis Analyses of Volatile Compounds

**Figure 7:**
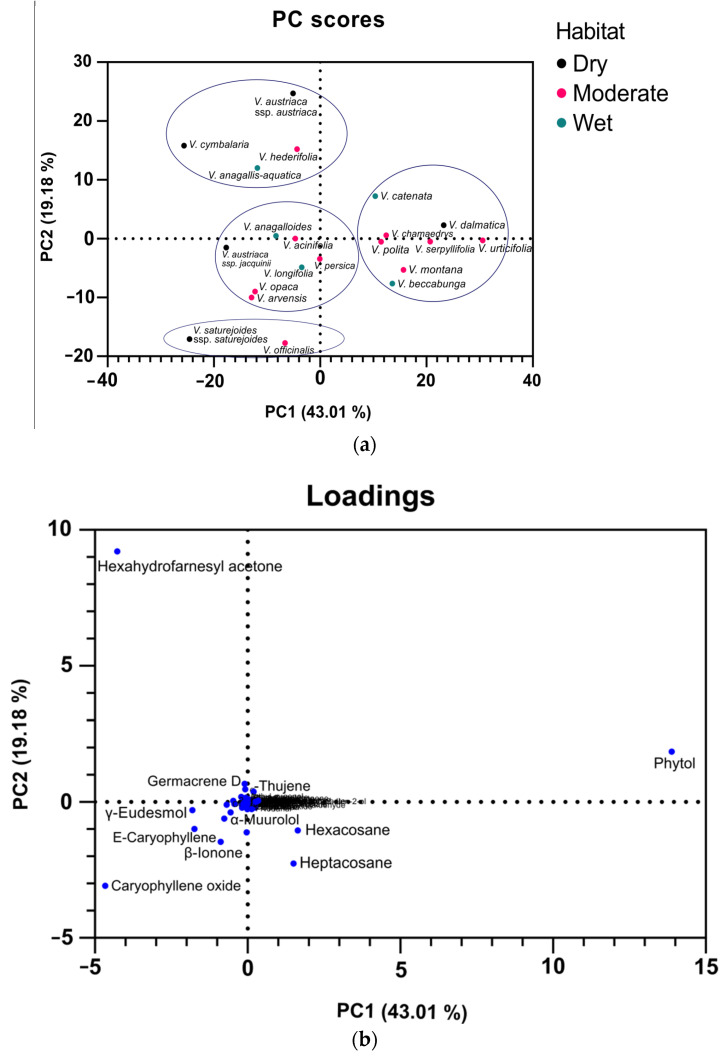
PCA analyses of volatile compounds of 21 *Veronica* species obtained by hydrodistillation. The PCA score plot allocating different species to clusters (**a**); PCA loading plots of volatiles from the first and second principal components (**b**).

**Figure 8:**
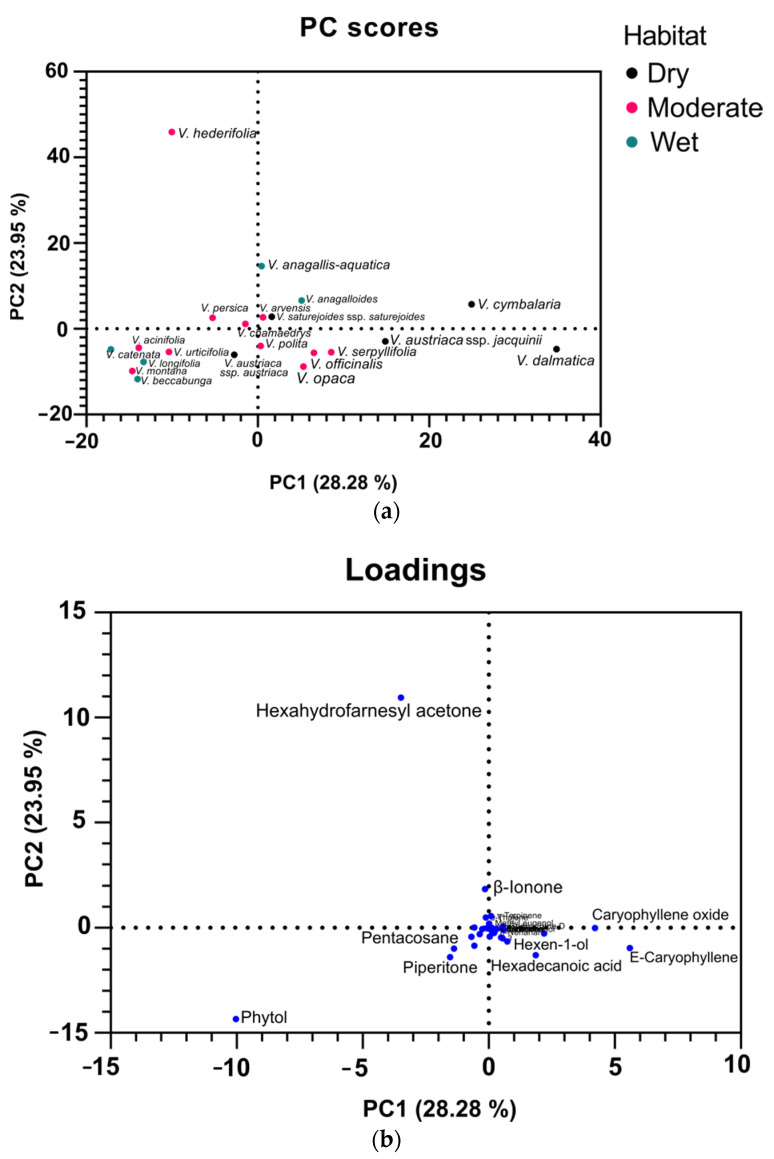
PCA analyses of volatile compounds of 21 *Veronica* species obtained by microwave extraction. The PCA score plot allocating different species to clusters (**a**); PCA loading plots of volatiles from the first and second principal components (**b**).

Principal Component Analysis (PCA) analyses were performed for VCs with amounts greater than 2%. Separate analyses were performed for the classical (Figure 7) and microwave (Figure 8) extraction methods. Principal Component (PC)1 and PC2 for VCs from the Clevenger extraction explained 62.19% of the variance and distinguished four clusters. The first cluster consists of *V. austriaca* ssp. *austriaca*, *V. cymbalaria*, *V. hederifolia*, and *V. anagallis-aquatica* (Figure 7a). All of these species are characterized by a high relative content of hexahydrofarnesyl acetone (27.17–39.77%). This component is located in the negative region of PC1 and the positive region of PC2 (Figure 7b). The second cluster consists of *V. anagalloide*s, *V. austriaca* ssp. *jacquinii*, *V. acinifolia*, *V. persica*, *V. longifolia*, *V. opaca*, and *V. arvensis*. These species are located around the center of the PCA score plot. They are all characterized by a moderate relative amount of hexadecanoic acid, hexahydrofarnesyl acetone, and phytol. The third cluster consists of *V. catenata*, *V. dalmatica*, *V. chamaedrys*, *V. polita*, *V. serpyllifolia*, *V. urticifolia*, *V. montana*, and *V. beccabunga*. The component that differentiates this cluster is phytol, which is located in the positive region of PC1 and PC2 (Figure 7b). The fourth cluster consists of two species: *V. saturejoides* ssp. *saturejoides* and *V. officinalis*. *V. saturejoides* ssp. *saturejoides* is characterized by a high relative concentration of caryophyllene oxide (34.53%). On the other hand, *V. officinalis* is characterized by a higher relative abundance of β-ionone, hexadecanoic acid, and heptacosane.

PC1 and PC2 for volatile compounds obtained by microwave extraction explained 51.23% of the variance. PCA analyses for volatile compounds obtained by microwave extraction gave no distinguishing features; however, we can point out some similarities among species (Figure 8). Species that are in the negative region of PC1 and PC2 include *V. catenata*, *V. urticifolia*, *V. montana*, *V. beccabunga*, *V. acinifolia,* and *V. longifolia* (Figure 8a). All of these species are characterized by a high relative concentration of phytol (24.21–47.55%). This component is located in the negative region of PC1 and PC2 (Figure 8b). Species located around the center of the PCA score plot include *V. anagalloide*s, *V. polita, V. persica*, *V. arvensis, V. saturejoides* ssp. *saturejoides*, *V. chamaedrys*, *V*. *austriaca* ssp. *austriaca*, *V*. *serpyllifolia*, *V*. *officinalis*, *V*. *opaca*, and *V*. *austriaca* ssp. *jacquinii*. These species are all characterized by a moderate relative concentration of hexadecanoic acid, hexahydrofarnesyl acetone, and phytol. *V. hederifolia* was allocated out of all the species because of its very high relative percentage of hexahydrofarnesyl acetone (59.15%). *V. cymbalaria* has a high concentration of caryophyllene oxide and *V. dalmatica* has a high concentration of *E*-caryophyllene, so they were allocated out of all the other clusters.

## 3. Discussion

The genus *Veronica* has undergone major changes in taxonomy over the last 20 years. It used to belong to the Scrophulariaceae family and then was transferred to the Plantaginaceae family following new genetic studies [1,2,23]. Albach and Taskova and their associates studied the iridoid glycosides of the genus *Veronica* and concluded that the distribution of these substances in the different species of the genus is consistent with the molecular phylogeny of the genus, thus showing that the chemistry of the genus can serve as a good indicator of interspecies and intergenus connections [10,11,24,25]. Chemosystematics (chemophenetics [26]) has been used throughout history to identify plants and other organisms and divide them into those that are suitable for use as food and those that should be avoided. One of the first researchers in this field was Greshoff (1909), who concluded that researchers in chemistry and botany should work together to study the plant world [27]. Taskova et al. isolated 16 iridoid glycosides from the genus *Veronica* and established a link between the chemical composition and the basic chromosome number [28]. The analysis of four *Veronica* species (*V. persica*, *V. polita*, *V. francispetae* M. A. Fisch., and *V. siaretensis* E. Lehm.) showed a qualitatively constant composition of iridoid samples in all species, regardless of environmental conditions [29]. This is consistent with the results of this study, because some major constituents were found to be present in all species regardless of the habitat in which they live. These VCs are hexahydrofarnesyl acetone, phytol, and hexadecanoic acid. The most abundant iridoids in the genus *Veronica* are generally aucubin and catalpol [28,30,31]. Albach et al. investigated the iridoid glycosides aucubine and catalpol in the genus *Veronica* and the plant species of *Paederota lutea* Scop. and concluded that the genera *Veronica* and *Paederota* are related based on the composition of these compounds [32]. This proves that iridoids are a very good marker of the chemophenetics of plant species. In our study, the VCs in *Veronica* species were studied to determine whether some of the compounds could be used as chemophenetic markers for future research and to determine whether different habitats affect the composition of these compounds.

Dunkić et al. [33] investigated the composition of the essential oil of *Veronica spicata* L. and identified the predominant hydrocarbons (heptacosane and pentacosane). The other predominant compounds were the diterpenes phytol and isophytol, the oxygenated monoterpenes piperitone and piperitone oxide, and aliphatic ketones [33]. In the Bulgarian species *Veronica officinalis*, a GC-MS study on ethanolic extracts of the aerial parts gave following composition: terpenes (hexahydrofarnesyl acetone), saturated and unsaturated fatty acids and esters, steroids, *p*-hydroxyphenylethyl alcohol, maltol, and loliolid [34]. Ertas et al. investigated the phytochemical composition of *Veronica thymoides* P. H. Davis subsp. *pseudocinerea* M. A. Fischer, concluded that the major component of the essential oil of this species is hexatriacontene, and found that the most abundant fatty acids in this plant were linoleic acid and hexadecanoic acid [14]. Feng Li investigated the composition of the essential oil of *Veronica linariifolia* Pall. ex Link and found that the major constituents were cyclohexene (25.83%), β-pinene (11.61%), 1S-α-pinene (10.65%), β-phellandrene (10.49%), β-myrcene (10.42%), and germacrene D (4.99%) (monoterpene and sesquiterpene hydrocarbons) [18]. If we compare all these results, then we can see that some constituents, such as hexadecanoic acid, hexahydrofarnesyl acetone, phytol, and different hydrocarbons, were found very frequently in all studies. These results may suggest that these VCs could be used as chemophenetic markers for the genus *Veronica*. Looking at the clusters for both Clevenger and microwave-assisted extraction, we can see that humidity-based habitats do not affect the composition of volatile compounds, as all habitats are represented in most clusters. Numerous other experiments have shown that VCs can be used to discriminate between species and cultivars [35,36,37,38].

Regarding the relative content of volatile compounds in *Veronica* taxa collected from dry habitats, oxygenated sesquiterpenes form the main class of classically isolated compounds. The exception is the endemic species *V. dalmatica*, in which the proportion of oxygenated sesquiterpenes was similar for both isolation methods. In the species *V. dalmatica*, oxygenated diterpenes were the most abundant in HD and sesquiterpene hydrocarbons were dominant in green extraction (Figure 2). In the group of plants collected from wet habitats, the percentages of compound classes extracted by both methods were the same. The greatest variation was found in the identification percentage of oxygenated monoterpenes in *V. beccabunga* (Figure 4). The compounds in the composition of monoterpene hydrocarbons were generally the least identified and the most isolated in the species of moderate habitats *V. hederifolia* by both methods of isolation (Figure 6). The composition of *V. officinalis* was found to be dominated by a group of compounds consisting of acids, alcohols, and esters. One of these compounds was the alcohol 3-hexen-1-ol, which was isolated by green extraction. This component is known to be one of the most important in the composition of VCs [39]. This species is often used in herbal tea, so the presence of 3-hexen-1-ol was expected as this compound is widely found in fresh tea leaves [39]. With this study on free VCs, we have increased our knowledge of the specialized metabolites that form the basis for further biological research.

Comparing the results from the extraction of VCs using classical hydrodistillation and microwave distillation, it can be seen that same main components were isolated by both methods but in different relative percentages. Some compounds were only isolated with either hydrodistillation or microwave distillation. This is logical as it is known that the process of hydrodistillation can negatively affect some of the compounds that are being decomposed due to high temperatures and long extraction times. On the other hand, microwave distillation can sometimes result in the isolation of fewer components as is stated in the study by Wu et al. [40]. They concluded in their research that hydrodistillation remains a better option for free VC extraction as it extracts the highest number of VCs. Looking at the results for the isolated VCs for the genus *Veronica* and the fact that all main compounds were extracted with both methods, microwave extraction should be considered when extracting VCs from a smaller amount (laboratory extraction) of sample because it is a greener choice that uses less water and energy and will not overheat the sample. In future analyses of *Veronica* species, it would be useful to investigate the composition of free VCs in water extracts (hydrosols) and compare the results of VC clustering with clustering based on genetic studies to identify potential chemophenetic (phytotaxonomic) markers among VCs.

## 4. Materials and Methods

### 4.1. Plant Material Collection and Preparation

**Figure 9:**
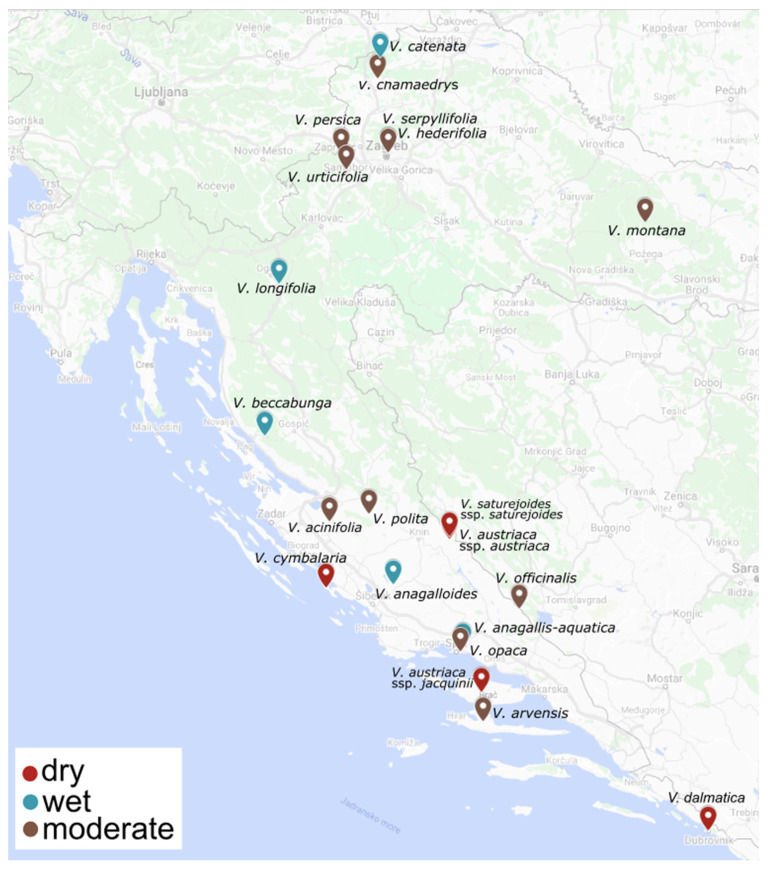
Map of the sites at which the studied *Veronica* taxa were collected.

Plant material was collected from March to July 2021 at different locations in Croatia (Table 1; Figure 9). All plant species were in the flowering stage. Voucher specimens were deposited at the Laboratory of Botany herbarium (HPMF-HR), Faculty of Science, University of Split, Croatia. All samples were air dried in a single layer and protected from direct sunlight for ten days.

Dried aboveground parts of the plant leaves, stems, and flowers (30–50 g) for each plant species were subjected to two different extraction methods: hydrodistillation and microwave-assisted extraction. The extracts, which were collected in pentane and diethyl ether (VWR, Radnor, PA, USA) from both extraction methods for all plant species, were dried over anhydrous sodium sulphate and stored at −20 °C until analysis.

### 4.2. Isolation of Volatile Compounds

#### 4.2.1. Classical Isolation

The VCs were isolated by hydrodistillation in a Clevenger-type apparatus (Šurlan, Medulin, Croatia) for 2.5 h using 30–50 g of dried plant material. In the inner tube of the Clevenger apparatus, VCs of the investigated species were collected in a mixed solution of pentane and diethyl ether (2:1).

#### 4.2.2. Green Isolation

Dried plant material (30–50 g for each plant species) was hydrated for 1 h before the isolation process. A Milestone ‘ETHOS X’ microwave laboratory oven (1900 W maximum) was used for microwave-assisted isolation. This oven is a 2.45 GHz multimode microwave reactor.

Regarding microwave-assisted distillation, a typical experiment was conducted at atmospheric pressure for 40 min at 500 W (98 °C). The distillation process started after 10 min. The distillate was collected in a side-tube using a pentane/diethyl ether trap, dried over anhydrous sodium sulphate, and stored at −20 °C until analysis.

### 4.3. GC and GC-MS Analyses

The above-described extracts were analyzed with a mass spectrometer (model 2100T; Varian Inc.) and a VF-5-ms non-polar capillary column (30 m with gas chromatography and mass spectrometry (GC-MS)) according to the method described in [6,19,21]. GC was performed by a gas chromatograph (model 3900, Varian Inc., Lake Forest, CA, USA) that was equipped with a flame ionization detector (FID), a mass spectrometer (model 2100T; Varian Inc.), a VF-5ms non-polar capillary column (inside diameter, 30 m × 0.25 mm; coating thickness, 0.25 μm; Palo Alto, CA, USA), and a CP-Wax 52 CB polar capillary column (i.d., 30 m × 0.25 mm; coating thickness, 0.25 μm; Palo Alto, CA, USA). The chromatographic conditions for the analysis of VCs were an FID detector temperature of 300 °C and an injector temperature of 250 °C. The gas carrier was helium at 1 mL min^−1^. The conditions for the VF-5-ms column were a temperature of 60 °C (isothermal) for 3 min, which was then increased to 246 °C at a rate of 3 °C min^−1^ and held (isothermal) for 25 min. The conditions for the CP Wax 52 column were a temperature of 70 °C (isothermal) for 5 min, which was then increased to 240 °C at a rate of 3 °C min^−1^ and held (isothermal) for 25 min. The injected volume was 2 μL and the split ratio was 1:20. The MS conditions were: ion source temperature, 200 °C; ionization voltage, 70 eV; mass scan range, 40–350 mass units [33]. The individual peaks for all samples were identified by a comparison of their retention indices of *n*-alkanes to those of authentic samples and the studies [41,42], a comparison to our libraries from previous work, and a comparison to other previously published material for *Veronica* species [14,18,34]. The results are expressed as the mean value of three analyses with the standard deviation.

### 4.4. PCA Analyses

Statistical analysis was performed in GraphPad Prism Version 9 (GraphPad Software, San Diego, CA, USA). All data in the tables are expressed as the mean ± SD (*n* = 3). Data included in the PCA analyses were obtained from the GC-MS analyses. PCA analyses were performed for VCs with amounts greater than 2%.

## 5. Conclusions

The results of this study show that hexahydrofarnesyl acetone, hexadecanoic acid, phytol, *E*-caryophyllene, and caryophyllene oxide are the major components identified by the classical (hydrodistillation) and green (microwave) methods of extraction regardless of the habitat of the 21 Croatian *Veronica* species studied. As these compounds were isolated in all species, they could be considered chemophenetic markers. Future research comparing clusters based on VCs and clusters resulting from genetic investigations might confirm this hypothesis. Looking at the results for the isolated VCs for the genus *Veronica* and the fact that all main compounds were extracted with both methods, microwave extraction should be considered when extracting VCs because it is a greener choice that uses less water and energy.

## Figures and Tables

**Table 1:** Details of the data collection and origin of the investigated *Veronica* taxa.

Taxa	Habitat	Locality	Latitude	Longitude	Altitude a.s.l. (m)	Voucher No.
*V. austriaca* ssp. *austriaca*	dry	Dinara Mt	44°02′20.1″ N	16°23′22.5″ E	1550	CROVeS-01-2021
*V. austriaca* ssp. *jacquini*i	dry	Brač Island	43°19′07.3″ N	16°36′08.5″ E	564	CROVeS-02-2021
*V. cymbalaria*	dry	Murter Island	43°48′36.6″ N	15°35′07.4″ E	37	CROVeS-03-2021
*V. dalmatica*	dry	Dubrovnik	42°39′19.1″ N	18°04′56.9″ E	58	CROVeS-04-2021
*V. saturejoides* ssp. *saturejoides*	dry	Dinara Mt	44°03′11.3″ N	16°23′29.7″ E	1697	CROVeS-05-2021
*V. anagallis-aquatica* ssp. *anagallis*-*aquatica*	wet	Split	43°31′43.5″ N	16°28′45.2″ E	22	CROVeS-06-2021
*V. anagalloides*	wet	Čikola River	43°49′36.2″ N	16°01′19.4″ E	45	CROVeS-07-2021
*V. beccabunga*	wet	Baške Oštarije	44°31′32.1″ N	15°10′34.2″ E	908	CROVeS-08-2021
*V. catenata*	wet	Trakoščan	46°15′30.3″ N	15°56′25.2″ E	240	CROVeS-09-2021
*V. longifolia*	wet	Oštarije	45°13′36.1″ N	15°16′18.2″ E	311	CROVeS-10-2021
*V. acinifolia*	moderate	Donji Karin	44°07′18.1″ N	15°36′13.7″ E	119	CROVeS-11-2021
*V. arvensis*	moderate	Hvar Island	43°10′42.3″ N	16°36′43.6″ E	38	CROVeS-12-2021
*V. chamaedrys*	moderate	Radoboj	46°09′49.4″ N	15°55′36.1″ E	260	CROVeS-13-2021
*V. hederifolia*	moderate	Zagreb	45°49′40.4″ N	15°58′59.6″ E	192	CROVeS-14-2021
*V. montana*	moderate	Papuk Mt	45°30′38.1″ N	17°39′57.2″ E	761	CROVeS-15-2021
*V. officinalis*	moderate	Kamešnica Mt	43°42′38.7″ N	16°50′47.9″ E	1225	CROVeS-16-2021
*V. opaca*	moderate	Split	43°30′32.3″ N	16°27′54.5″ E	67	CROVeS-17-2021
*V. persica*	moderate	Samoborsko gorje	45°49′41.6″ N	15°40′32.9″ E	301	CROVeS-18-2021
*V. polita*	moderate	Kaštel Žegarski	44°09′26.1″ N	15°51′56.0″ E	53	CROVeS-19-2021
*V. serpyllifolia*	moderate	Zagreb	45°49′40.3″ N	15°58′59.5″ E	192	CROVeS-20-2021
*V. urticifolia*	moderate	Plešivica Mt	45°45′05.7″ N	15°42′28.3″ E	350	CROVeS-21-2021

## Data Availability

All data is contained within the article and Supplementary Materials.

## Supplementary Materials

The following supporting information can be downloaded at: https://www.mdpi.com/article/10.3390/plants11070902/s1, Table S1. Chemical composition of the VCs obtained by hydro-distillation from the aerial parts of Veronica taxa collected on dry habitats, Table S2. Chemical composition of the VCs obtained by microwave extraction from the aerial parts of Veronica taxa collected on dry habitats, Table S3. Chemical composition of the VCs obtained by hydro-distillation from the aerial parts of Veronica taxa collected on wet habitats, Table S4. Chemical composition of the VCs obtained by microwave extraction from the aerial parts of Veronica taxa collected on wet habitats, Table S5. Chemical composition of the VCs obtained by hydro-distillation from the aerial parts of Veronica taxa collected on moderate habitats, Table S6. Chemical composition of the VCs obtained by microwave extraction from the aerial parts of Veronica taxa collected on moderate habitats.
