# Supplementary material for: Hydrodistillation and Microwave Extraction of Volatile Compounds: Comparing Data for Twenty-One Veronica Species from Different Habitats
Source: Plants (Basel). 2022 Mar 28;11(7):902. doi: 10.3390/plants11070902 (PMC9002677; doi:10.3390/plants11070902)
Supplement: Supplementary file 1 [file plants-11-00902-s001.zip › Veronica_Supplementary Table 1.pdf]

Table S1. Chemical composition of the VCs obtained by hydro-distillation from the aerial parts of *Veronica* taxa collected on dry habitats.

			V. austriaca ssp. *austriaca*	*V. austriaca* ssp. *jacquinii*	*V. cymbalaria*	*V. dalmatica.*	*V. saturejoides* ssp. *saturejoides*
Component	RI[a]	RI[b]	VC±SD	VC±SD	VC±SD	VC±SD	VC±SD
**Monoterpene hydrocarbons**			**0.94**	**-**	**-**	**0.15**	**0.88**
α-Thujene	924	1012	0.94±0.01				
α-Pinene*	935	1017	-	-	-	-	0.88±0.01
β -Phellandrene	1002	1195	-	-	-	0.15±0.03	-
**Oxygenated monoterpenes**			**1.09**	**-**	**1.97**	**15.06**	**1.79**
1,8-Cineole	1026	1210	0.83±0.01	-	-	0.76±0.01	-
γ-Terpinene	1057	1225	-	-	-	2.61±0.01	-
Linalool	1095	1506	0.26±0.01	-	0.26±0.01	4.72±0.01	0.89±0.05
allo-Ocimene	1128	1390	-	-	0.22±0.15	-	-
Camphor	1151	1499	-	-	-	0.72±0.01	-
Terpinen-4-ol	1174	1686	-	-	0.22±0.01	-	-
Borneol	1176	1719	-	-	-	1.59±0.01	-
α-Terpineol	1184	1660	-	-	-	3.08±0.03	0.28±0.01
trans-p-Mentha-*1(7),8*-dien-2-ol	1187	1803	-	-	0.62±0.01	-	0.62±0.02
Menthyl acetate	1294	1550	-	-	0.65±0.01	1.58±0.02	-
**Sesquiterpene hydrocarbons**			**7.76**	**8.01**	**10.91**	**13.55**	**16.86**
α-Copaene	1377	1484	-	-	0.78±0.01	-	1.53±0.01
E-Caryophyllene*	1424	1585	1.23±0.01	8.01±0.01	3.95 ±0.04	3.48±0.01	9.43±0.01
allo-Aromadendrene	1465	1662	-	-	1.32±0.01	2.59±0.01	1.47±0.01

β-Chamigrene	1478	1724	-	-	-	0.27±0.12	-
Germacrene D	1481	1692	5.28±0.01	-	1.42±0.01	3.87±0.02	2.61±0.01
δ-Selinene	1492	1756	1.25±0.01	-	2.32±0.01	3.34±0.01	-
δ-Cadinene	1517	1745	-	-	1.12±0.01	-	1.82±0.01
**Oxygenated sesquiterpenes**			**44.63**	**31.1**	**60.16**	**13.46**	**52.05**
Spathulenol	1577	2101	-	-	-	0.25±0.01	1.8±0.01
Caryophyllene oxide*	1581	1955	4.86±0.01	13.98±0.01	10.92±0.01	0.52±0.01	34.53±0.01
Viridiflorol	1592	2099	-	-	-	-	1.05±0.01
γ-Eudesmol	1632	2175	-	-	9.61±0.01	-	0.2±0.03
α-Muurolol	1645	2181	-	-	3.30±0.01	2.38±0.01	7.06±0.01
α-Bisabolol	1685	2210	-	-	-	2.59±0.03	-
α-Bisabolol oxide	1748	2511	-	-	-	-	0.53±0.01
Hexahydrofarnesyl acetone*	1839	2113	39.77±0.01	17.12±0.01	36.33±0.01	7.72±0.13	6.88±0.01
**Oxygenated diterpene**			**20.12**	**4.58**	**-**	**41.22**	**-**
Phytol*	1942	2610	20.12±0.01	4.58±0.01	-	41.22±0.01	-
**Phenolic compounds**			**-**	**0.33**	**1.38**	**0.85**	**-**
Thymol*	1289	2154	**-**	**-**	0.82±0.01	**-**	**-**
p-Vinyl guaicol	1313	2156	-	-	0.27±0.03	-	-
Thymol acetate	1349	-	**-**	**-**	**-**	0.85±0.01	**-**
Methyl eugenol	1403	2005	-	0.33±0.01	**-**	**-**	**-**
(Z)-Methyl isoeugenol	1451	2070	**-**	**-**	0.29±0.01	**-**	**-**
**Acids, alcohols and esters**			**13.38**	**44.19**	**12.94**	**8.24**	**23.92**
Isopentyl acetate	863	1127	-	-	-	0.24±0.09	0.28±0.01

Benzaldehyde	952	1508	-	-	0.98±0.01	1.25±0.01	3.98±0.01
Benzene acetaldehyde	1036	1633	-	-	0.48±0.01	-	1.12±0.01
n-Nonanal	1100	1389	-	-	0.89±0.02	-	1.68±0.01
Hexyl 2-methyl butanoate	1233	1425	-	-	-	0.21±0.01	-
n-Decanol	1266	1711	-	-	0.11±0.05	-	0.98±0.01
2,4-Decadienal	1304	1764	2.23±0.01	-	-	-	4.25±0.01
(E)-*β*-Damascenone	1384	1819	-	-	-	0.69±0.01	-
β-Ionone	1487	1935	-	-	7.28±0.01	3.27±0.01	3.98±0.01
Benzyl benzoate	1760	2613	-	-	1.56±0.01	-	-
1-Hexadecanol	1874	2371	-	-	0.89±0.01	1.45±0.01	1.51±0.03
Hexadecanoic acid*	1959	2912	11.15±0.01	32.17±0.01	0.75±0.01	1.13±0.01	6.14±0.01
Oleic acid	2133	2998	-	12.02±0.01	-	-	-
Octadecanol acetate	2209	-	-	0.98±0.01	-	-	-
**Hydrocarbons**			**8.35**	**5.94**	**5.89**	**6.96**	**1.12**
Eicosane*	2000	2000	-	1.39±0.01	1.24±0.01	-	1.12±0.01
Heneicosane*	2100	2100	-	0.83±0.01	0.98±0.17	0.87±0.01	-
Docosane*	2200	2200	-	2.69±0.01	2.13±0.01	2.11±0.01	-
Tricosane*	2300	2300	-	-	-	1.96±0.01	-
Tetracosane*	2400	2400	-	-	0.83±0.01	1.77±0.01	-
Pentacosane*	2500	2500	3.92±0.01	1.03±0.01	0.71±0.04	0.25±0.03	-
Heptacosane*	2700	2700	3.74±0.01	-	-	-	-
Octacosane*	2800	2800	0.69±0.01	-	-	-	-
**Total identification (%)**			**96.27**	**94.15**	**93.25**	**92.53**	**96.62**

Retention indices (RIs) were determined relative to a series of n-alkanes (C8–C40) on capillary columns VF5-ms (RI[a]) and CPWax 52 (RI[b]); Identification method: RI, comparison of RIs with those in a self-generated library reported in the literature [41] and/or with authentic samples; comparison of mass spectra with those in the NIST02 and Wiley 9 mass spectral libraries; *co-injection with reference compounds; -, not identified; SD, standard deviation of triplicate analysis.
