# Supplementary material for: Hydrodistillation and Microwave Extraction of Volatile Compounds: Comparing Data for Twenty-One Veronica Species from Different Habitats
Source: Plants (Basel). 2022 Mar 28;11(7):902. doi: 10.3390/plants11070902 (PMC9002677; doi:10.3390/plants11070902)
Supplement: Supplementary file 1 [file plants-11-00902-s001.zip › Veronica_Supplementary Table 2.pdf]

Table S2. Chemical composition of the VCs obtained by microwave extraction from the aerial parts of *Veronica* taxa collected on dry habitats.

			V. austriaca ssp. *austriaca*	*V. austriaca* ssp. *jacquinii*	*V. cymbalaria*	*V. dalmatica*	*V. saturejoides* ssp. *saturejoides*
Component	RI[a]	RI[b]	VC±SD	VC±SD	VC±SD	VC±SD	VC±SD
**Monoterpene hydrocarbons**			**1.69**	**1.93**	**-**	**-**	**1.18**
α-Thujene	924	1012	1.69±0.01	-	-	-	-
α-Pinene*	935	1017	-	1.93±0.01	-	-	1.18±0.01
**Oxygenated monoterpenes**			**-**	**0.87**	**1.62**	**3**	**4.6**
Linalool	1095	1506	-	0.87±0.01	0.96±0.15	1.22±0.01	1.78±0.02
Camphor	1151	1499	-	-	-	-	0.52±0.01
α-Terpineol	1184	1660	-	-	-	1.78±0.13	0.88±0.01
trans-p-Mentha-*1(7),8*-dien-2-ol	1187	1803	-	-	-	-	1.42±0.03
Menthyl acetate	1294	1550	-	-	0.66±0.07	-	-
**Sesquiterpene hydrocarbons**			**6.01**	**2.78**	**13.7**	**57.8**	**17.43**
α-Copaene	1377	1484	0.57±0.03	-	-	-	0.89±0.03
E-Caryophyllene*	1424	1585	0.66±0.08	1.79±0.01	6.13 ±0.01	39.53±0.01	8.49±0.01
allo-Aromadendrene	1465	1662	-	-	2.28±0.01	15.43±0.01	-
Germacrene D	1481	1692	1.01±0.01	0.68±0.01	2.34±0.03	1.89±0.01	5.11±0.01
δ-Selinene	1492	1756	3.77±0.01	0.31±0.1	1.52±0.01	0.95±0.01	1.73±0.01
δ-Cadinene	1517	1745	-	-	1.43±0.01	-	1.21±0.01
**Oxygenated sesquiterpenes**			**12.74**	**15.25**	**47.69**	**15.72**	**29.05**
Spathulenol	1577	2101	-	-	-	4.76±0.01	2.25±0.04
Caryophyllene oxide*	1581	1955	2.97±0.01	6.64±0.01	32.72±0.01	7.52±0.15	8.43±0.01

Viridiflorol	1592	2099	-	-	-	-	0.65±0.02
γ-Eudesmol	1632	2175	-	-	0.61±0.07	-	-
α-Bisabolol	1685	2210	-	-	1.01±0.01	-	-
Hexahydrofarnesyl acetone*	1839	2113	9.77±0.01	8.61±0.01	13.35±0.01	3.44±0.02	17.72±0.01
**Oxygenated diterpene**			**24.21**	**6.58**	**3.71**	**2.75**	**22.47**
Phytol*	1942	2610	24.21±0.01	6.58±0.01	3.71±0.01	2.75±0.02	22.47±0.01
**Phenolic compounds**			**-**	**-**	**1.44**	**-**	**-**
Thymol*	1289	2154	-	-	0.45±0.01	-	-
(Z)-Methyl isoeugenol	1451	2070	-	-	0.99±0.16	-	-
**Acids, alcohols and esters**			**7.72**	**24.99**	**26.21**	**12.28**	**16.21**
Isopentyl acetate	863	1127	-	-	-	-	0.56±0.09
Benzaldehyde	952	1508	2.37±0.01	-	1.24±0.01	-	1.48±0.01
Benzene acetaldehyde	1036	1633	-	-	0.42±0.01	0.75±0.01	1.02±0.01
n-Nonanal	1100	1389	0.83±0.02	-	-	1.68±0.01	1.14±0.18
Hexyl 2-methyl butanoate	1233	1425	-	-	-	0.91±0.01	-
n-Decanol	1266	1711	-	1.52±0.05	-	-	1.88±0.01
2,4-Decadienal	1304	1764	-	-	-	-	0.55±0.01
(E)-*β*-Damascenone	1384	1819	-	-	-	0.29±0.06	-
β-Ionone	1487	1935	-	1.01±0.03	7.88±0.01	6.00±0.01	1.84±0.03
Benzyl benzoate	1760	2613	-	-	-	-	-
1-Hexadecanol	1874	2371	-	-	0.64±0.13	-	1.1±0.01
Hexadecanoic acid*	1959	2912	4.15±0.01	22.17±0.01	15.72±0.01	2.65±0.01	6.64±0.01
Oleic acid	2133	2998	0.37±0.01	0.29±0.01	0.31±0.01	-	-

**Hydrocarbons**			**40.65**	**41.99**	**2.5**	**5.46**	**5.86**
Eicosane*	2000	2000	1.84±0.01	1.12±0.01	0.24±0.01	1.51±0.04	0.12±0.01
Heneicosane*	2100	2100	0.77±0.04	0.44±0.02	0.88±0.01	0.87±0.01	2.89±0.01
Docosane*	2200	2200	1.17±0.05	1.63±0.1	-	0.48±0.06	1.59±0.01
Tricosane*	2300	2300	13.61±0.01	17.32±0.01	-	1.13±0.01	0.43±0.03
Tetracosane*	2400	2400	3.65±0.01	1.61±0.05	0.89±0.01	1.12±0.01	0.35±0.07
Pentacosane*	2500	2500	1.37±0.02	5.91±0.01	0.49±0.05	0.35±0.23	0.48±0.01
Hexacosane*	2600	2600	9.29±0.01	8.97±0.01	-	-	-
Heptacosane*	2700	2700	8.95±0.02	4.11±0.15	-	-	-
Octacosane*	2800	2800	-	0.88±0.01	-	-	-
**Total identification (%)**			**93.02**	**94.39**	**96.87**	**97.01**	**96.8**

Retention indices (RIs) were determined relative to a series of n-alkanes (C8–C40) on capillary columns VF5-ms (RI[a]) and CPWax 52 (RI[b]); Identification method: RI, comparison of RIs with those in a self-generated library reported in the literature [41] and/or with authentic samples; comparison of mass spectra with those in the NIST02 and Wiley 9 mass spectral libraries; *co-injection with reference compounds; -, not identified; SD, standard deviation of triplicate analysis.
