# Supplementary material for: Hydrodistillation and Microwave Extraction of Volatile Compounds: Comparing Data for Twenty-One Veronica Species from Different Habitats
Source: Plants (Basel). 2022 Mar 28;11(7):902. doi: 10.3390/plants11070902 (PMC9002677; doi:10.3390/plants11070902)
Supplement: Supplementary file 1 [file plants-11-00902-s001.zip › Veronica_Supplementary Table 3.pdf]

Table S3. Chemical composition of the VCs obtained by hydro-distillation from the aerial parts of *Veronica* taxa collected on wet habitats.

			*V. anagallis-aqua*tica	*V. anagalloides*	*V. beccabunga*	*V. catenata*	*V. longifolia*
Component	RI[a]	RI[b]	VC±SD	VC±SD	VC±SD	VC±SD	VC±SD
**Monoterpene hydrocarbons**			**1.42**	**-**	**-**	**-**	**-**
β -Phellandrene	1002	1195	1.42±0.01	-	-	-	-
**Oxygenated monoterpenes**			**5.75**	**5.57**	**8.33**	**-**	**3.02**
1,8-Cineole	1026	1210	1.83±0.01	-	-	-	-
γ-Terpinene	1057	1225	0.49±0.05	2.53±0.01	-	-	-
Linalool	1095	1506	1.21±0.01	1.71±0.01	-	-	0.65±0.01
Terpinen-4-ol	1174	1686	-	0.68±0.1	-	-	-
α-Terpineol	1184	1660	-	-	-	-	0.82±0.07
trans-p-Mentha-*1(7),8*-dien-2-ol	1187	1803	2.22±0.01	0.65±0.04	1.85±0.01	-	1.55±0.01
Piperitone	1250	1719	-	-	5.79±0.01		
Menthyl acetate	1294	1550	-	-	0.69±0.01	-	-
**Sesquiterpene hydrocarbons**			**10.27**	**11.59**	**3.89**	**12.55**	**17.3**
α-Copaene	1377	1484	-	-	-	-	-
E-Caryophyllene*	1424	1585	5.49±0.01	4.07±0.01	2.75 ±0.01	4.11±0.02	4.13±0.01
allo-Aromadendrene	1465	1662	1.10±0.03	2.00±0.01	0.72±0.01	1.23±0.01	2.17±0.01
β-Chamigrene	1478	1724	-	-	-	-	0.29±0.01
Germacrene D	1481	1692	1.28±0.01	2.22±0.15	0.42±0.01	3.87±0.02	4.61±0.01
δ-Selinene	1492	1756	0.65±0.01	1.15±0.01	-	3.34±0.01	3.75±0.01
δ-Cadinene	1517	1745	1.75±0.01	2.15±0.1	-	-	2.35±0.01
**Oxygenated sesquiterpenes**			**32.18**	**24.47**	**11.06**	**19.82**	**19.52**

Spathulenol	1577	2101	0.65±0.01	2.85±0.01	-	0.55±0.01	2.56±0.01
Caryophyllene oxide*	1581	1955	4.36±0.01	4.91±0.01	4.22±0.01	1.55±0.01	5.58±0.01
Viridiflorol	1592	2099	-	-	0.60±0.16	-	1.75±0.01
γ-Eudesmol	1632	2175	-	-	0.11±0.01	-	-
α-Muurolol	1645	2181	-	-	-	-	0.55±0.01
α-Bisabolol	1685	2210	-	1.66±0.01	-	-	-
α-Bisabolol oxide	1748	2511	-	0.72±0.09	-	-	-
Hexahydrofarnesyl acetone*	1839	2113	27.17±0.01	14.33±0.01	6.13±0.01	17.75±0.02	9.08±0.01
**Oxygenated diterpene**			**9.42**	**9.58**	**27.31**	**29.92**	**13.63**
Phytol*	1942	2610	9.42±0.01	9.58±0.01	27.31±0.01	29.92±0.01	13.63±0.01
**Phenolic compounds**			**7.42**	**5.27**	**0.30**	**-**	**-**
Thymol*	1289	2154	-	-	-	-	-
p-Vinyl guaicol	1313	2156	2.46±0.01	-	-	-	-
Methyl eugenol	1403	2005	4.96±0.01	2.66±0.04	0.30±0.01	-	-
(Z)-Methyl isoeugenol	1451	2070	-	2.61±0.01	-	-	-
**Acids, alcohols and esters**			**20.98**	**31.14**	**8.97**	**16.29**	**27.57**
Isopentyl acetate	863	1127	-	-	-	-	-
Benzaldehyde	952	1508	2.78±0.01	-	-	0.25±0.01	0.91±0.01
Benzene acetaldehyde	1036	1633	4.27±0.01	6.62±0.01	1.58±0.01	-	1.19±0.01
n-Nonanal	1100	1389	-	3.08±0.01	2.85±0.02	1.65±0.04	8.18±0.01
Hexyl 2-methyl butanoate	1233	1425	-	2.03±0.03	-	-	-
n-Decanol	1266	1711	0.68±0.01	-	-	-	0.45±0.01
2,4-Decadienal	1304	1764	2.23±0.01	-	-	-	-

(E)-β-Damascenone	1384	1819	1.96±0.01	3.78±0.01	-	-	-
β-Ionone	1487	1935	4.41±0.01	1.62±0.01	1.28±0.01	4.37±0.01	7.10±0.01
Benzyl benzoate	1760	2613	-	-	0.56±0.15	-	-
Hexadecanoic acid*	1959	2912	4.65±0.01	13.67±0.01	2.72±0.01	10.02±0.01	9.74±0.01
Oleic acid	2133	2998	-	0.34±0.03	-	-	-
**Hydrocarbons**			**7.54**	**5.92**	**36.62**	**16.22**	**15.78**
Eicosane*	2000	2000	-	-	1.64±0.01	-	-
Heneicosane*	2100	2100	-	-	0.98±0.17	.	-
Docosane*	2200	2200	3.72±0.01	3.91±0.01	0.13±0.01	3.11±0.03	4.32±0.07
Tricosane*	2300	2300	1.69±0.04	-	1.18±0.02	4.16±0.01	1.28±0.15
Tetracosane*	2400	2400	0.35±0.02	-	0.83±0.01	3.79±0.01	1.29±0.01
Pentacosane*	2500	2500	-	2.01±0.01	0.51±0.04	0.28±0.1	6.81±0.01
Hexacosane*	2600	2600	-	-	16.21±0.01	-	2.08±0.17
Heptacosane*	2700	2700	0178±0.02	-	14.89±0.01	4.88±0.04	-
Octacosane*	2800	2800	-	-	0.25±0.02	-	-
**Total identification (%)**			**94.98**	**93.54**	**96.78**	**94.8**	**96.82**

Retention indices (RIs) were determined relative to a series of n-alkanes (C8–C40) on capillary columns VF5-ms (RI^a) and CPWax 52 (RI^b); Identification method: RI, comparison of RIs with those in a self-generated library reported in the literature [41] and/or with authentic samples; comparison of mass spectra with those in the NIST02 and Wiley 9 mass spectral libraries; *co-injection with reference compounds; -, not identified; SD, standard deviation of triplicate analysis.
