# Supplementary material for: Hydrodistillation and Microwave Extraction of Volatile Compounds: Comparing Data for Twenty-One Veronica Species from Different Habitats
Source: Plants (Basel). 2022 Mar 28;11(7):902. doi: 10.3390/plants11070902 (PMC9002677; doi:10.3390/plants11070902)
Supplement: Supplementary file 1 [file plants-11-00902-s001.zip › Veronica_Supplementary Table 4.pdf]

Table S4. Chemical composition of the VCs obtained by microwave extraction from the aerial parts of *Veronica* taxa collected on wet habitats.

			*V. anagallis-aqua*tica	*V. anagalloides*	*V. beccabunga*	*V. catenata*	*V. longifolia*
Component	RI[a]	RI[b]	VC±SD	VC±SD	VC±SD	VC±SD	VC±SD
**Oxygenated monoterpenes**			**12.52**	**4.82**	**30.1**	**-**	**-**
1,8-Cineole	1026	1210	0.46±0.01	-	-	-	-
γ-Terpinene	1057	1225	7.58±0.01	3..81±0.01	-	-	-
Linalool	1095	1506	0.71±0.01	-	-	-	-
Camphor	1151	1499	0.91±0.01	-	-	-	-
Terpinen-4-ol	1174	1686	-	1.01±0.04	-	-	-
trans-p-Mentha-*1(7),8*-dien-2-ol	1187	1803	1.78±0.01	-	0.82±0.01	-	-
Piperitone	1250	1719	-	-	29.28±0.01	-	-
Menthyl acetate	1294	1550	1.08±0.01	-	-	-	-
**Sesquiterpene hydrocarbons**			**5.1**	**10.16**	**3.69**	**2.67**	**2.98**
E-Caryophyllene*	1424	1585	3.29±0.01	4.01±0.01	2.95 ±0.04	2.48±0.01	1.43±0.01
allo-Aromadendrene	1465	1662	-	2.11±0.01	0.32±0.02	0.19±0.01	1.55±0.01
Germacrene D	1481	1692	0.88±0.01	3.07±0.01	0.42±0.01	-	-
δ-Selinene	1492	1756	0.22±0.04	0.97±0.01	-	-	-
δ-Cadinene	1517	1745	0.71±0.01	-	-	-	-
**Oxygenated sesquiterpenes**			**29.45**	**31.19**	**11.18**	**24.39**	**12.37**
Spathulenol	1577	2101	0.65±0.01	-	-	0.65±0.1	0.81±0.01
Caryophyllene oxide*	1581	1955	2.55±0.01	8.58±0.01	1.62±0.01	6.52±0.01	1.53±0.01
Viridiflorol	1592	2099	-	0.81±0.01	-	-	0.75±0.01
α-Bisabolol	1685	2210	0.28±0.01	1.91±0.01	-	-	-

α-Bisabolol oxide	1748	2511	-	0.77±0.02	-	-	-
Hexahydrofarnesyl acetone*	1839	2113	25.97±0.01	19.12±0.01	9.56±0.01	17.22±0.01	9.28±0.01
**Oxygenated diterpene**			**14.56**	**14.88**	**34.54**	**42.26**	**37.18**
Phytol*	1942	2610	14.56±0.01	14.88±0.01	34.54±0.01	42.26±0.01	37.18±0.01
**Phenolic compounds**			**3.43**	**0.33**	**-**	**-**	**-**
Methyl eugenol	1403	2005	3.43±0.01	0.33±0.07	**-**	**-**	**-**
**Acids, alcohols and esters**			**25.93**	**18.17**	**9.54**	**12.98**	**30.01**
Isopentyl acetate	863	1127	-	-	-	-	0.22±0.03
Benzaldehyde	952	1508	1.63±0.01	-	-	-	-
Benzene acetaldehyde	1036	1633	9.67±0.01	-	2.48±0.01	-	4.17±0.01
n-Nonanal	1100	1389	1.82±0.01	2.12±0.01	0.89±0.02	-	2.68±0.01
Hexyl 2-methyl butanoate	1233	1425	-	0.44±0.01	-	-	-
n-Decanol	1266	1711	1.28±0.01	-	-	-	0.58±0.01
2,4-Decadienal	1304	1764	0.28±0.05	-	-	-	-
(E)-β-Damascenone	1384	1819	0.12±0.16	0.52±0.07	-	0.32±0.09	-
β-Ionone	1487	1935	6.36±0.01	4.22±0.01	1.43±0.01	0.27±0.01	16.22±0.01
Hexadecanoic acid*	1959	2912	4.77±0.01	9.17±0.01	4.74±0.01	5.81±0.01	6.14±0.01
Oleic acid	2133	2998	-	1.02±0.01	-	2.25±0.01	-
Octadecanol acetate	2209	-	-	0.68±0.03	-	4.33±0.01	-
**Hydrocarbons**			**5.3**	**18.12**	**8.26**	**15.1**	**10.71**
Eicosane*	2000	2000	0.72±0.01	1.12±0.01	-	-	-
Heneicosane*	2100	2100	0.18±0.01	0.33±0.04	-	-	-
Docosane*	2200	2200	2.22±0.01	5.62±0.01	4.15±0.01	-	2.82±0.01

Tricosane*	2300	2300	0.75±0.01	-	-	3.03±0.01	-
Tetracosane*	2400	2400	1.15±0.01	-	-	12.07±0.01	-
Pentacosane*	2500	2500	-	5.43±0.01	1.13±0.01	-	4.71±0.01
Hexacosane*	2600	2600	-	3.90±0.01	0.27±0.09	-	1.48±0.01
Heptacosane*	2700	2700	0.28±0.1	0.95±0.01	1.45±0.01	-	1.70±0.01
Octacosane*	2800	2800	-	0.77±0.15	1.26±0.01	-	-
**Total identification (%)**			**97.29**	**96.79**	**97.31**	**97.4**	**93.25**

Retention indices (RIs) were determined relative to a series of n-alkanes (C8–C40) on capillary columns VF5-ms (RI[a]) and CPWax 52 (RI[b]); Identification method: RI, comparison of RIs with those in a self-generated library reported in the literature [41] and/or with authentic samples; comparison of mass spectra with those in the NIST02 and Wiley 9 mass spectral libraries; *co-injection with reference compounds; -, not identified; SD, standard deviation of triplicate analysis.
