# Supplementary material for: Hydrodistillation and Microwave Extraction of Volatile Compounds: Comparing Data for Twenty-One Veronica Species from Different Habitats
Source: Plants (Basel). 2022 Mar 28;11(7):902. doi: 10.3390/plants11070902 (PMC9002677; doi:10.3390/plants11070902)
Supplement: Supplementary file 1 [file plants-11-00902-s001.zip › Veronica_Supplementary Table 5.pdf]

**Table S5.** Chemical composition of the VCs obtained by hydro-distillation from the aerial parts of *Veronica* taxa collected on moderate habitats.

| | | | *V. acinifolia* | *V. arvensis* | *V. chamaedrys* | *V. hederifolia* | *V. montana* | *V. officinalis* |
|---|---|---|---|---|---|---|---|---|
| Component | RI[a] | RI[b] | VC±SD | VC±SD | VC±SD | VC±SD | VC±SD | VC±SD |
| **Monoterpene hydrocarbons** | | | **-** | **-** | **-** | **4.86** | **-** | **-** |
| α-Thujene | 924 | 1012 | - | - | - | 4.86±0.01 | - | - |
| **Oxygenated monoterpenes** | | | **0.75** | **1.2** | **3.26** | **0.98** | **-** | **-** |
| Terpinen-4-ol | 1174 | 1686 | 0.75±0.1 | - | 1.49±0.01 | - | - | - |
| α-Terpineol | 1184 | 1660 | - | - | 0.92±0.01 | 0.98±0.15 | - | - |
| *trans-p*-Mentha-*1(7),8*-dien-2-ol | 1187 | 1803 | - | 0.52±0.01 | 0.85±0.01 | - | - | - |
| Hexyl 2-methyl butanoate | 1233 | 1425 | - | 0.68±0.05 | - | - | - | - |
| **Sesquiterpene hydrocarbons** | | | **6.73** | **12.44** | **5.58** | **9.19** | **0.7** | **3.77** |
| *E*-Caryophyllene* | 1424 | 1585 | 4.46±0.01 | 6.21±0.01 | 2.43 ±0.01 | 4.11±0.01 | 0.13±0.01 | 3.12±0.01 |
| *allo*-Aromadendrene | 1465 | 1662 | 0.89±0.05 | 0.85±0.01 | 0.82±0.01 | 2.26±0.01 | 0.57±0.01 | 0.65±0.01 |
| β-Chamigrene | 1478 | 1724 | - | 0.77±0.03 | - | - | - | - |
| Germacrene D | 1481 | 1692 | 0.43±0.01 | 1.25±0.03 | 1.02±0.01 | 1.47±0.03 | - | - |
| δ-Selinene | 1492 | 1756 | 0.95±0.01 | 2.01±0.01 | 1.31±0.15 | 1.35±0.04 | - | - |
| δ-Cadinene | 1517 | 1745 | - | 1.35±0.01 | - | - | - | - |
| **Oxygenated sesquiterpenes** | | | **28.92** | **40.44** | **18.54** | **37.18** | **14.14** | **12.93** |
| Spathulenol | 1577 | 2101 | 0.95±0.01 | - | - | 0.58±0.01 | - | |
| Caryophyllene oxide* | 1581 | 1955 | 7.71±0.01 | 14.11±0.01 | 6.25±0.01 | 4.59±0.01 | 7.28±0.01 | 4.65±0.01 |
| Viridiflorol | 1592 | 2099 | - | - | 0.91±0.03 | - | - | |
| γ-Eudesmol | 1632 | 2175 | - | 19.98±0.01 | 0.56±0.01 | 3.16±0.01 | - | 0.88±0.01 |
| α-Muurolol | 1645 | 2181 | - | - | - | - | - | 2.38±0.01 |

| | | | | | | | | |
|---|---|---|---|---|---|---|---|---|
| $\alpha$-Cadinol | 1655 | 2208 | - | - | - | - | - | 0.72±0.01 |
| $\alpha$-Bisabolol | 1685 | 2210 | 2.12±0.01 | - | - | - | - | 0.38±0.01 |
| $\alpha$-Bisabolol oxide | 1748 | 2511 | 2.77±0.01 | - | - | - | - | 0.69±0.01 |
| Hexahydrofarnesyl acetone* | 1839 | 2113 | 15.37±0.01 | 6.35±0.01 | 10.82±0.01 | 28.85±0.01 | 6.86±0.01 | 3.25±0.02 |
| **Oxygenated diterpene** | | | **15.63** | **7.54** | **31.66** | **18.53** | **32.61** | **6.72** |
| Phytol* | 1942 | 2610 | 15.63±0.01 | 7.54±0.01 | 31.66±0.01 | 18.53±0.01 | 32.61±0.01 | 6.72±0.01 |
| **Phenolic compounds** | | | **6.9** | **6.99** | **4.16** | **3.25** | **-** | **-** |
| Thymol* | 1289 | 2154 | - | - | - | 0.56±0.1 | - | - |
| *p*-Vinyl guaicol | 1313 | 2156 | 2.41±0.01 | 3.11±0.01 | 2.64±0.01 | 2.22±0.01 | - | - |
| Thymol acetate | 1349 | - | 2.38±0.01 | - | - | - | - | - |
| Methyl eugenol | 1403 | 2005 | 2.11±0.01 | 2.16±0.02 | 1.52±0.01 | 0.47±0.1 | - | - |
| *(Z)*-Methyl isoeugenol | 1451 | 2070 | - | 1.72±0.01 | - | - | - | - |
| **Acids, alcohols and esters** | | | **35.73** | **19.44** | **24.44** | **18.84** | **16.6** | **31.97** |
| Isopentyl acetate | 863 | 1127 | - | - | 0.56±0.01 | - | - | - |
| Benzaldehyde | 952 | 1508 | 0.32±0.13 | - | 1.32±0.01 | 3.24±0.01 | 1.84±0.01 | - |
| *2*-Pentyl furan | 984 | 1230 | - | 4.52±0.01 | - | - | - | - |
| Benzene acetaldehyde | 1036 | 1633 | 3.97±0.01 | 2.69±0.01 | 7.28±0.01 | - | 3.34±0.01 | - |
| *n*-Nonanal | 1100 | 1389 | 2.18±0.01 | 2.91±0.01 | 1.81±0.1 | 2.71±0.01 | - | - |
| Hexyl 2-methyl butanoate | 1233 | 1425 | 2.45±0.01 | - | - | - | - | - |
| n-Decanol | 1266 | 1711 | 1.88±0.01 | 0.85±0.01 | - | - | - | - |
| *2,4*-Decadienal | 1304 | 1764 | 1.45±0.01 | - | - | - | - | - |
| *(E)*-$\beta$-Damascenone | 1384 | 1819 | 3.12±0.01 | 2.61±0.01 | 1.22±0.07 | 2.57±0.01 | - | 0.42±0.03 |
| $\beta$-Ionone | 1487 | 1935 | 17.01±0.01 | 2.69±0.01 | 4.41±0.01 | 3.07±0.01 | 2.18±0.01 | 18.11±0.01 |

| | | | | | | | | |
|---|---|---|---|---|---|---|---|---|
| Benzyl benzoate | 1760 | 2613 | - | - | 2.11±0.01 | - | - | - |
| Hexadecanoic acid* | 1959 | 2912 | 3.35±0.01 | 3.17±0.01 | 5.73±0.01 | 7.25±0.01 | 9.24±0.01 | 13.21±0.01 |
| Oleic acid | 2133 | 2998 | - | - | - | - | - | 0.23±0.07 |
| Octadecanol acetate | 2209 | - | - | - | - | - | - | 1.11±0.01 |
| **Hydrocarbons** | | | **0.22** | **4.45** | **6.75** | **3.45** | **32.9** | **38.49** |
| Eicosane* | 2000 | 2000 | - | - | - | - | 0.31±0.01 | - |
| Heneicosane* | 2100 | 2100 | - | 0.88±0.01 | 0.21±0.1 | . | 0.42±0.07 | 0.34±0.05 |
| Docosane* | 2200 | 2200 | 0.22±0.01 | 1.32±0.01 | 3.14±0.01 | 0.81±0.02 | 3.34±0.02 | 2.15±0.01 |
| Tricosane* | 2300 | 2300 | - | 0.65±0.01 | 0.32±0.03 | 0.46±0.01 | 0.53±0.01 | 0.65±0.07 |
| Tetracosane* | 2400 | 2400 | - | 0.89±0.01 | 2.83±0.01 | 0.77±0.01 | 0.45±0.01 | 0.92±0.01 |
| Pentacosane* | 2500 | 2500 | - | 0.71±0.01 | 0.56±0.01 | 0.21±0.03 | 10.47±0.01 | 11.89±0.01 |
| Hexacosane* | 2600 | 2600 | - | - | 0.22±0.05 | 0.88±0.15 | 12.45±0.01 | 1.52±0.01 |
| Heptacosane* | 2700 | 2700 | - | - | 2.30±0.01 | 0.32±0.05 | 3.82±0.01 | 20.67±0.01 |
| Octacosane* | 2800 | 2800 | - | - | - | - | 1.11±0.01 | 0.35±0.04 |
| **Total identification (%)** | | | **94.88** | **92.5** | **94.39** | **96.28** | **96.95** | **93.88** |

**Table 5.** Continue

| | | | *V. opaca* | *V. persica* | *V. polita* | *V. serpyllifolia* | *V. urticifolia* |
|---|---|---|---|---|---|---|---|
| Component | RI[a] | RI[b] | VC±SD | VC±SD | VC±SD | VC±SD | VC±SD |
| **Oxygenated monoterpenes** | | | **0.63** | **-** | **-** | **0.68** | **-** |
| Linalool | 1095 | 1506 | 0.42±0.01 | - | - | - | - |
| Terpinen-4-ol | 1174 | 1686 | 0.21±0.04 | - | - | - | - |
| $\alpha$-Terpineol | 1184 | 1660 | - | - | - | 0.68±0.01 | - |

| | | | | | | | |
|---|---|---|---|---|---|---|---|
| **Sesquiterpene hydrocarbons** | | | **11.56** | **17.68** | **10.36** | **4.91** | **0.6** |
| *α*-Copaene | 1377 | 1484 | - | - | - | - | - |
| *E*-Caryophyllene* | 1424 | 1585 | 11.23±0.01 | 9.29±0.01 | 6.57 ±0.01 | 2.11±0.01 | 0.43±0.01 |
| *allo*-Aromadendrene | 1465 | 1662 | - | 2.81±0.01 | 0.42±0.01 | 0.28±0.01 | 0.17±0.04 |
| *β*-Chamigrene | 1478 | 1724 | - | 2.02±0.01 | - | - | - |
| Germacrene D | 1481 | 1692 | 0.33±0.01 | 0.75±0.01 | 1.06±0.03 | 0.67±0.01 | - |
| *δ*-Selinene | 1492 | 1756 | - | 2.81±0.01 | 2.31±0.04 | 1.85±0.02 | - |
| **Oxygenated sesquiterpenes** | | | **35.86** | **26.34** | **20.18** | **13.2** | **7.34** |
| Spathulenol | 1577 | 2101 | - | - | - | 0.88±0.01 | - |
| Caryophyllene oxide* | 1581 | 1955 | 11.75±0.01 | 10.11±0.01 | 7.55±0.01 | 4.19±0.01 | 0.38±0.01 |
| Viridiflorol | 1592 | 2099 | - | - | 0.87±0.08 | - | - |
| *γ*-Eudesmol | 1632 | 2175 | 4.62±0.01 | 0.72±0.01 | 0.29±0.01 | 0.21±0.01 | - |
| *α*-Muurolol | 1645 | 2181 | 1.73±0.01 | 1.71±0.01 | - | - | - |
| *α*-Cadinol | 1655 | 2208 | 1.06±0.01 | - | - | - | - |
| *α*-Bisabolol | 1685 | 2210 | 4.02±0.01 | 1.23±0.01 | 0.71±0.01 | - | - |
| *α*-Bisabolol oxide | 1748 | 2511 | 1.21±0.01 | 2.26±0.01 | 0.48±0.01 | - | - |
| Hexahydrofarnesyl acetone* | 1839 | 2113 | 11.47±0.01 | 10.31±0.01 | 10.28±0.01 | 7.92±0.01 | 6.96±0.01 |
| **Oxygenated diterpene** | | | **6.68** | **20.21** | **31.18** | **39.79** | **47.55** |
| Phytol* | 1942 | 2610 | 6.68±0.01 | 20.21±0.01 | 31.18±0.01 | 39.79±0.01 | 47.55±0.01 |
| **Phenolic compounds** | | | **0.75** | **5.33** | **2.61** | **2.09** | **-** |
| *p*-Vinyl guaicol | 1313 | 2156 | 0.75±0.01 | 3.14±0.01 | 2.26±0.01 | 0.52±0.01 | - |
| Methyl eugenol | 1403 | 2005 | - | 1.42±0.01 | 0.35±0.01 | 1.57±0.04 | **-** |
| *(Z)*-Methyl isoeugenol | 1451 | 2070 | **-** | 0.77±0.01 | - | **-** | **-** |

| | | | | | | | |
|---|---|---|---|---|---|---|---|
| **Acids, alcohols and esters** | | | **13.04** | **20.2** | **18.21** | **28.54** | **15.83** |
| Benzaldehyde | 952 | 1508 | 0.29±0.02 | 0.75±0.01 | 5.47±0.01 | 0.29±0.01 | - |
| Benzene acetaldehyde | 1036 | 1633 | 0.45±0.01 | 1.65±0.01 | 1.45±0.01 | 3.92±0.01 | 0.74±0.01 |
| *n*-Nonanal | 1100 | 1389 | 0.21±0.01 | 0.76±0.01 | 0.51±0.1 | 2.78±0.01 | 1.15±0.01 |
| n-Decanol | 1266 | 1711 | - | 1.08±0.01 | 0.43±0.01 | 0.96±0.01 | - |
| *2,4*-Decadienal | 1304 | 1764 | - | 0.44±0.07 | - | - | - |
| *(E)-β*-Damascenone | 1384 | 1819 | - | 2.57±0.01 | 0.32±0.04 | 0.77±0.01 | 2.14±0.01 |
| *β*-Ionone | 1487 | 1935 | 7.51±0.01 | 5.60±0.01 | 3.28±0.01 | 7.54±0.01 | 0.56±0.01 |
| 1-Hexadecanol | 1874 | 2371 | - | - | - | - | - |
| Hexadecanoic acid* | 1959 | 2912 | 4.58±0.01 | 7.35±0.01 | 6.75±0.01 | 12.28±0.01 | 11.24±0.01 |
| **Hydrocarbons** | | | **28.91** | **4.38** | **10.64** | **7.79** | **21.43** |
| Eicosane* | 2000 | 2000 | 0.66±0.05 | - | 0.42±0.05 | - | 0.39±0.08 |
| Heneicosane* | 2100 | 2100 | 0.79±0.01 | 0.37±0.01 | 0.21±0.03 | . | 0.49±0.01 |
| Docosane* | 2200 | 2200 | 7.45±0.01 | 3.10±0.01 | 3.87±0.01 | 2.01±0.01 | 3.33±0.01 |
| Tricosane* | 2300 | 2300 | 1.71±0.01 | 0.25±0.01 | 0.37±0.02 | 0.48±0.04 | 1.21±0.01 |
| Tetracosane* | 2400 | 2400 | 2.11±0.01 | - | 0.81±0.01 | 0.77±0.01 | 0.25±0.01 |
| Pentacosane* | 2500 | 2500 | 4.14±0.01 | - | 0.36±0.01 | 0.98±0.01 | 0.48±0.01 |
| Hexacosane* | 2600 | 2600 | 0.34±0.01 | - | 4.27±0.05 | 3.13±0.01 | 0.32±0.05 |
| Heptacosane* | 2700 | 2700 | 11.71±0.01 | 0.21±0.01 | 0.33±0.01 | 0.42±0.03 | 13.82±0.01 |
| Octacosane* | 2800 | 2800 | - | 0.45±0.01 | - | - | 2.14±0.01 |
| **Total identification (%)** | | | **97.43** | **94.14** | **93.18** | **97** | **93.15** |

Retention indices (RIs) were determined relative to a series of n-alkanes (C8–C40) on capillary columns VF5-ms ($RI^a$) and CPWax 52 ($RI^b$); Identification method: RI, comparison of RIs with those in a self-generated library reported in the literature [41] and/or with authentic samples; comparison of mass spectra with those in the NIST02 and Wiley 9 mass spectral libraries; *co-injection with reference compounds; -, not identified; SD, standard deviation of triplicate analysis.
