# Supplementary material for: Hydrodistillation and Microwave Extraction of Volatile Compounds: Comparing Data for Twenty-One Veronica Species from Different Habitats
Source: Plants (Basel). 2022 Mar 28;11(7):902. doi: 10.3390/plants11070902 (PMC9002677; doi:10.3390/plants11070902)
Supplement: Supplementary file 1 [file plants-11-00902-s001.zip › Veronica_Supplementary Table 6.pdf]

Table S6. Chemical composition of the VCs obtained by microwave extraction from the aerial parts of *Veronica* taxa collected on moderate habitats.

			V. acinifolia	*V. arvensis*	*V. chamaedrys*	*V. hederifolia*	*V. montana*	*V. officinalis*
Component	RI[a]	RI[b]	VC±SD	VC±SD	VC±SD	VC±SD	VC±SD	VC±SD
**Monoterpene hydrocarbons**			**-**	**1.57**	**-**	**2.82**	**-**	**-**
α-Thujene	924	1012	-	-	-	2.82±0.01	-	-
β -Phellandrene	1002	1195	-	1.57±0.01	-	-	-	-
**Oxygenated monoterpenes**			**-**	**0.93**	**0.76**	**-**	**-**	**-**
Terpinen-4-ol	1174	1686	-	-	0.76±0.01	-	-	-
trans-p-Mentha-*1(7),8*-dien-2-ol	1187	1803	-	0.93±0.01	-	-	-	-
**Sesquiterpene hydrocarbons**			**8.55**	**9.66**	**2.36**	**1.26**	**0.44**	**4.77**
E-Caryophyllene*	1424	1585	6.51±0.01	3.25±0.01	1.05 ±0.01	1.10±0.01	0.44±0.01	3.12±0.01
allo-Aromadendrene	1465	1662	0.21±0.07	1.81±0.01	0.82±0.01	0.16±0.01	-	1.65±0.01
Germacrene D	1481	1692	1.83±0.01	2.45±0.01	0.12±0.01	-	-	-
δ-Selinene	1492	1756	-	1.22±0.01	0.37±0.01	-	-	-
δ-Cadinene	1517	1745	-	0.93±0.01	-	-	-	-
**Oxygenated sesquiterpenes**			**23.99**	**26.98**	**18.8**	**59.66**	**13.64**	**12.04**
Spathulenol	1577	2101	0.25±0.01	-	-	-	-	
Caryophyllene oxide*	1581	1955	5.52±0.01	7.11±0.01	1.22±0.01	0.51±0.06	2.61±0.01	4.15±0.01
Viridiflorol	1592	2099	-	-	0.12±0.03	-	-	
γ-Eudesmol	1632	2175	-	2.32±0.01	0.77±0.01	-	-	-
α-Cadinol	1655	2208	-	-	-	-	0.69±0.01	-
α-Bisabolol	1685	2210	1.78±0.01	-	-	-	0.72±0.01	0.22±0.01
α-Bisabolol oxide	1748	2511	0.27±0.01	-	-	-	0.45±0.01	0.85±0.01

Hexahydrofarnesyl acetone*	1839	2113	16.17±0.01	17.55±0.01	16.69±0.01	59.15±0.01	9.17±0.01	6.82±0.01
**Oxygenated diterpene**			**39.88**	**22.57**	**18.88**	**14.58**	**37.03**	**16.89**
Phytol*	1942	2610	39.88±0.01	22.57±0.01	18.88±0.01	14.58±0.01	37.03±0.01	16.89±0.01
**Phenolic compounds**			**1.06**	**1.97**	**2.12**	**-**	**-**	**-**
p-Vinyl guaicol	1313	2156	0.58±0.01	1.51±0.01	0.84±0.01	-	-	-
Methyl eugenol	1403	2005	0.48±0.01	0.46±0.06	0.57±0.01	-	-	-
(Z)-Methyl isoeugenol	1451	2070	-	-	0.71±0.01	-	-	-
**Acids, alcohols and esters**			**11.45**	**26.71**	**22.16**	**16.6**	**17.51**	**52.59**
Isopentyl acetate	863	1127	-	-	-	-	-	1.04±0.01
3-Hexen-1-ol	873	1383		-	-	-	-	22.04±0.01
Benzaldehyde	952	1508	-	3.12±0.01	-	-	0.91±0.04	-
2-Pentyl furan	984	1230	-	0.51±0.01	-	-	-	-
Benzene acetaldehyde	1036	1633	-	1.24±0.01	3.22±0.01	-	2.31±0.01	-
n-Nonanal	1100	1389	1.46±0.01	2.31±0.01	0.85±0.1	-	-	6.72±0.01
Hexyl 2-methyl butanoate	1233	1425	0.24±0.01	-	0.75±0.1	-	-	1.61±0.01
n-Decanol	1266	1711	0.45±0.01	-	0.32±0.1	-	-	-
2,4-Decadienal	1304	1764	0.53±0.01	-	-	-	-	-
(E)-*β*-Damascenone	1384	1819	0.16±0.01	0.82±0.01	0.42±0.01	-	0.31±0.01	0.99±0.03
β-Ionone	1487	1935	4.09±0.01	7.29±0.01	0.77±0.01	15.03±0.01	7.68±0.01	7.79±0.01
Hexadecanoic acid*	1959	2912	4.52±0.01	17.42±0.01	15.83±0.01	1.57±0.01	5.81±0.01	12.40±0.01
**Hydrocarbons**			**7.59**	**3.03**	**32.67**	**0.68**	**25.04**	**7.19**
Heneicosane*	2100	2100	-	0.58±0.07	-	.	1.48±0.07	-
Docosane*	2200	2200	1.42±0.01	1.68±0.01	12.13±0.01	0.68±0.01	3.13±0.02	0.92±0.01

Tricosane*	2300	2300	0.42±0.01	0.77±0.01	0.15±0.07	-	0.88±0.01	-
Tetracosane*	2400	2400	-	-	5.55±0.01	-	0.95±0.01	-
Pentacosane*	2500	2500	5.75±0.01	-	8.36±0.01	-	14.90±0.01	0.15±0.03
Hexacosane*	2600	2600	-	-	0.13±0.02	-	-	0.54±0.01
Heptacosane*	2700	2700	-	-	5.22±0.01	-	3.70±0.01	5.52±0.01
Octacosane*	2800	2800	-	-	1.13±0.01	-	-	0.06±0.01
**Total identification (%)**			**92.52**	**93.42**	**97.75**	**95.6**	**93.66**	**93.48**

Table 6. Continue

			V. opaca	*V. persica*	*V. polita*	*V. serpyllifolia*	*V. urticifolia*
Component	RI[a]	RI[b]	VC±SD	VC±SD	VC±SD	VC±SD	VC±SD
**Oxygenated monoterpenes**			**-**	**-**	**-**	**0.86**	**-**
Linalool	1095	1506	-	-	-	0.86±0.01	-
**Sesquiterpene hydrocarbons**			**11.55**	**6.08**	**6.03**	**14.4**	**0.69**
α-Copaene	1377	1484	-	-	-	1.61±0.01	-
β-Elemene	1389	1593	-	1.29±0.01	1.16±0.01	2.29±0.01	-
E-Caryophyllene*	1424	1585	8.62±0.01	2.62±0.01	4.17 ±0.01	6.83±0.01	0.14±0.01
allo-Aromadendrene	1465	1662	0.85±0.01	0.82±0.05	0.52±0.02	-	-
β-Chamigrene	1478	1724	-	0.17±0.01	-	-	-
Germacrene D	1481	1692	0.53±0.01	0.35±0.01	0.07±0.01	3.24±0.01	0.55±0.01
δ-Selinene	1492	1756	1.55±0.01	0.83±0.01	0.11±0.01	0.43±0.01	-
**Oxygenated sesquiterpenes**			**13.37**	**23.7**	**15.88**	**27.92**	**13.71**

Spathulenol	1577	2101	-	-	-	1.25±0.01	-
Caryophyllene oxide*	1581	1955	6.75±0.01	3.14±0.01	1.48±0.01	14.74±0.01	0.49±0.04
Viridiflorol	1592	2099	-	-	0.44±0.01	-	-
γ-Eudesmol	1632	2175	2.62±0.01	1.22±0.01	-	2.52±0.01	-
α-Muurolol	1645	2181	0.75±0.01	-	-	-	-
α-Cadinol	1655	2208	0.42±0.01	-	-	0.82±0.01	-
α-Bisabolol	1685	2210	2.02±0.01	0.63±0.01	1.41±0.01	1.21±0.01	-
α-Bisabolol oxide	1748	2511	0.81±0.03	0.24±0.07	1.73±0.01	0.84±0.01	-
Hexahydrofarnesyl acetone*	1839	2113	5.87±0.01	18.47±0.01	10.82±0.01	6.54±0.01	13.22±0.01
**Oxygenated diterpene**			**23.18**	**23.71**	**19.88**	**18.72**	**31.12**
Phytol*	1942	2610	23.18±0.01	23.71±0.01	19.88±0.01	18.72±0.01	31.12±0.01
**Phenolic compounds**			**-**	**1.7**	**0.45**	**2.89**	**-**
p-Vinyl guaicol	1313	2156	-	0.74±0.01	0.45±0.01	1.64±0.01	-
Methyl eugenol	1403	2005	-	0.95±0.01	-	1.25±0.01	-
**Acids, alcohols and esters**			**35.19**	**14.24**	**8.64**	**18.6**	**31.01**
Benzaldehyde	952	1508	1.16±0.01	0.39±0.01	-	0.85±0.01	0.49±0.01
1-Octen-3-ol	974	1433	-	-	-	-	1.67±0.01
Benzene acetaldehyde	1036	1633	2.09±0.01	2.05±0.01	0.46±0.01	1.25±0.01	7.65±0.03
n-Nonanal	1100	1389	1.64±0.01	1.06±0.02	0.51±0.01	1.28±0.01	0.42±0.05
2,4-Decadienal	1304	1764	-	0.34±0.03	-	-	-
(E)-β-Damascenone	1384	1819	2.51±0.01	0.55±0.01	0.65±0.01	1.56±0.01	0.16±0.01
β-Ionone	1487	1935	7.52±0.01	4.54±0.01	1.33±0.01	5.74±0.01	4.51±0.01
Benzyl benzoate	1760	2613	0.59±0.01	-	-	-	-

Hexadecanoic acid*	1959	2912	19.68±0.01	5.31±0.01	5.69±0.01	7.71±0.01	16.11±0.01
Oleic acid	2133	2998	-	-	-	0.21±0.01	-
**Hydrocarbons**			**9.2**	**26.61**	**42.53**	**10.05**	**21.26**
Eicosane*	2000	2000	0.29±0.15	1.51±0.01	0.30±0.01	-	-
Heneicosane*	2100	2100	0.59±0.02	0.32±0.05	0.13±0.15	-	.
Docosane*	2200	2200	3.48±0.01	2.85±0.01	2.45±0.01	4.20±0.01	2.76±0.01
Tricosane*	2300	2300	0.76±0.01	0.14±0.01	0.37±0.02	0.30±0.03	0.18±0.01
Tetracosane*	2400	2400	-	0.84±0.02	0.89±0.01	2.59±0.01	2.71±0.01
Pentacosane*	2500	2500	-	5.27±0.01	1.76±0.01	0.18±0.07	8.91±0.01
Hexacosane*	2600	2600	0.44±0.05	0.17±0.15	4.55±0.01	2.74±0.01	0.53±0.04
Heptacosane*	2700	2700	0.75±0.03	14.28±0.01	15.13±0.01	-	5.75±0.01
Octacosane*	2800	2800	2.89±0.01	1.23±0.01	16.95±0.02	-	0.42±0.05
**Total identification (%)**			**92.49**	**96.04**	**93.41**	**93.44**	**97.79**

Retention indices (RIs) were determined relative to a series of n-alkanes (C8–C40) on capillary columns VF5-ms (RI^a) and CPWax 52 (RI^b); Identification method: RI, comparison of RIs with those in a self-generated library reported in the literature [41] and/or with authentic samples; comparison of mass spectra with those in the NIST02 and Wiley 9 mass spectral libraries; *co-injection with reference compounds; -, not identified; SD, standard deviation of triplicate analysis.
